# The importance of education combined with tailored exercise in the health and wellness of older adults: a community case study

**DOI:** 10.3389/fpsyg.2024.1488903

**Published:** 2024-10-10

**Authors:** Mindy Brummett, Chassiti Oglesby, Sarah Barkus, Nina Meg Wheelock, Allison Tate

**Affiliations:** The Department of Physical Therapy, The University of North Texas Health Science Center, Fort Worth, TX, United States

**Keywords:** metabolic syndrome, older adults, physical activity, mental health, wellness

## Abstract

Current literature states the importance of mental and physical health in combating the effects of metabolic syndrome; however, there is limited information on whether providing education on the syndrome along with mental and individualized physical exercises improves perceived confidence in the older adult population. A solution to this problem would be to provide a course to this population with a primary goal of education and exercise prescription. A community case study was implemented in the spring of 2024 with the purpose of measuring perceived confidence in metabolic syndrome, management of stress and anxiety, and how to move safely with exercise. Twenty-nine older adults with an average age of 76.1 years were recruited from a local senior citizen center. A course was given to the participants that included education and prescription of exercises tailored to the needs of the individual. Before and after the course, participants completed a confidence survey investigating their confidence in lowering the risk for metabolic syndrome, managing stress/anxiety, and understanding how to move safely with exercise. Regarding the post surveys, knowing how to lower the risk of metabolic syndrome increased by 46%, learning how to manage stress and anxiety increased by 50%, and understanding how to exercise safely increased by 41%. The data from this study suggests that providing education along with specific exercise prescription improved the participant’s confidence in lowering their risk for metabolic syndrome, management of stress and anxiety, and how to move safely with exercise.

## 1 Introduction

As a person ages, numerous physiological changes occur increasing the susceptibility to metabolic syndrome. Research indicates that approximately 35% of adults are affected by metabolic syndrome, with the prevalence rising to 46.7% among individuals aged 60 years and above (Gallardo-Alfaro et al., 2019). Metabolic syndrome is a cluster of conditions that include central abdominal obesity, high blood pressure, elevated blood sugar levels, and abnormal cholesterol levels leading to poor overall health (Silva et al., 2019; Ekram et al., 2023; Gouveia et al., 2021). Metabolic syndrome is diagnosed clinically when an individual meets three or more of the following criteria: central abdominal obesity defined by a waist circumference exceeding 40 inches for men or 35 inches in women; high blood pressure, characterized by a systolic pressure of 130 mmHg or greater, or a diastolic pressure of 85 mmHg or higher; abnormal cholesterol levels, including triglyceride levels of 150 mg/dL or above and high-density lipoprotein cholesterol levels below 40 mg/dL in men or 50 mg/dL in women (Fahed et al., 2022). Due to the multifaceted nature of the syndrome, high costs in healthcare resources along with rising rates of morbidity in the older population are seen (Ekram et al., 2023; Rezende et al., 2014).

.To reduce the impact and medical burden of metabolic syndrome, education can be given regarding the condition along with prescription of mental and physical exercises. Mental exercises can be implemented via relaxation techniques. Relaxation techniques promote regulation of the cardiovascular, respiratory, and gastrointestinal systems through the modulation of the autonomic nervous system (Hamasaki, 2020). Diaphragmatic breathing is often utilized in relaxation exercises due to a significant increase in sustained mindfulness, lower cortisol levels, and decreased perceived negative emotions (Ma et al., 2017). Diaphragmatic breathing is a slow, deep breathing method that involves the contraction of the diaphragm, expansion of the belly, and deepening of inhalation and exhalation maneuvers (Hamasaki, 2020; Ma et al., 2017).

A sedentary lifestyle is commonly found in the older adult population, with reports of persons over 60 years old spending most of their day in sitting positions and other activities involving lower rates of metabolic energy expenditure (Pate et al., 2008; Matthews et al., 2008; Davis et al., 2001). Consequently, this population frequently fails to meet physical activity guidelines, leading to diminished strength, poor cardiovascular endurance, cognitive decline, and a heightened risk for falls, while also exacerbating the development of metabolic syndrome (Hallal et al., 2012; Rezende et al., 2014; Mora and Valencia, 2018; Xu et al., 2022).

Current literature states the importance of education, mental health, and physical activity guidelines in combating the syndrome’s effects; however, there is limited information on whether providing education combined with a prescription of individualized exercises can help this population (Lenze et al., 2022; Earnest et al., 2014; Stephens et al., 2020; Guerreiro et al., 2022). To alleviate the burden of metabolic syndrome in older adults, it is essential to offer education about the condition and ways to reduce its risk factors, preventing its onset or progression. Additionally, implementing diaphragmatic breathing and customizing exercise programs to suit individual tolerances can further modify the risk factors associated with the syndrome.

The purpose of this community case study was to measure the impact of education along with prescription of tailored exercise for management of metabolic syndrome in the older adult population. Based on previous literature, we hypothesized combining education, mental health practices, and personalized physical exercises would improve confidence levels in older adults (Lenze et al., 2022; Earnest et al., 2014; Stephens et al., 2020; Guerreiro et al., 2022).

## 2 Context

### 2.1 Recruitment

The North Texas Institutional Review Board (IRB) at the University of North Texas Health Science Center (# 2185389-1) approved this study. In the spring and summer of 2024, adults who were members of the Keller Senior Activities Center in Keller, Texas, were contacted through an online newsletter about a free class called “Mindful Motion.” This class was created by the investigators of the research study and was detailed in a description sent to the Director of the Senior Activities Center. The course, led by the investigators, aimed to educate participants on metabolic syndrome and its adverse health effects while providing prescription of mental and individualized physical exercises. Attendees were advised to wear comfortable clothes and tennis shoes.

### 2.2 Participants

Older adults were included if they fulfilled all the following criteria: (1) 55 years or older and (2) members of the Keller Senior Activities Center. Older adults were excluded if they fulfilled the following criteria: (1) not filling out an informed consent form or (2) could not attend the provided course. After consenting to the study, 29 participants were given a random participant ID number by one of the investigators. This number was then used for the demographic questionnaire and confidence surveys. The participant ID number ensured that the participants’ identities were blinded to the researchers, maintaining the anonymity of the participants throughout the study.

### 2.3 Quantitative and qualitative assessments

A demographic questionnaire gathered the person’s age, gender, number of days per week they exercised, fall history (how many falls and where they occurred), and current use of assistive devices. A systematic review and meta-analysis were referred to when questions for the questionnaire were utilized (Rezende et al., 2014; Xu et al., 2022; Guerreiro et al., 2022; Tsai et al., 2021; Lee et al., 2023). A pre and post confidence survey was used to measure the participant’s perceived confidence on how to lower the risk for metabolic syndrome, manage stress/anxiety, move safely when exercising, and understand how exercise can help with their health and well-being. The informed consent form, demographic survey, and confidence surveys took place on the day of the course before the course began. Upon completion of the entire course, a post-confidence survey was administered to assess whether education combined with customized exercise enhanced perceived confidence among the participants. The blinded investigators administered all forms and surveys pre and post intervention.

Please see Appendices A–C for the IRB consent form, demographic questionnaire, and confidence survey.

### 2.4 Program design and educational session

The literature has demonstrated the significance of providing education to older adults. A study involving community-dwelling adults aged 65 and older revealed significant improvements in health and functional measures, with lasting effects observed up to 48 weeks after the intervention (Uemura et al., 2021). Therefore, the class began with a 20-min power point presentation on metabolic syndrome. This was provided by one of the investigators. During this presentation, the overall condition was defined, followed by additional details describing each component of the syndrome. The aim of the educational session was to enhance the knowledge and understanding of the syndrome along with management of the modifiable risk factors.

Please see Appendix C for the educational presentation provided to the participants.

### 2.5 Program design and mental health exercise

Immediately after the educational session, diaphragmatic breathing techniques along with examples of mindfulness applications were introduced and practiced for the mental health exercise. This approach was chosen due to a single session of such breathing has been shown to alleviate symptoms of emotional exhaustion, anxiety, and depression (Laborde et al., 2022). Moreover, educating older adults on the use of various mindfulness applications on smartphone devices has been shown to significantly reduce self-measured systolic blood pressure and stress, along with improvements in overall well-being (Bostock et al., 2019).

While the participants sat comfortably in a chair, diaphragmatic breathing was demonstrated and practiced. One of the investigators led this 5-min mental health exercise. Instruction was provided to hold one hand on the chest and another on the abdomen while sitting tall in a chair, maintaining good posture. The participants were instructed to feel their stomach for expansion without chest compensation to perform this exercise effectively. Maintaining the manual contact, the participants then practiced taking deep breaths through the nose followed by a breath out as if blowing on a candle. Practicing of this technique occurred for 10 repetitions. After the practice time, the participants were then provided with a handout on how to perform the exercise at home.

Please see Appendix D for the mental health exercise handout provided to the participants.

### 2.6 Program design and individualized exercise prescription

Immediately following the relaxation exercise, the TUG (Timed up and go) test was shown by the investigators. This demonstration aimed for the participants to self-select which individualized exercise group (low-intensity or high-intensity) they would be involved in for the remainder of the class. This ensured that the exercise prescription would be specific to the needs of the participants.

The TUG test is valid and easily administered for the older adult population (Nightingale et al., 2019). The mean time for persons aged 60–69 is 8.1 s, whereas in those aged 70–79, the mean time is 9.2 s (Browne and Nair, 2019). Therefore, the investigators used a time of 10 s to determine which exercise group the participants should select. If participants felt they could perform a TUG test in less than 10 s, they were placed in the high-intensity interval training group. If participants thought they could perform a TUG test in over 10 s, they were placed in the low-intensity training group. The participants were then told to split into the groups they picked for 50 min and were allowed to ask any questions they had throughout each session.

### 2.7 Program design and high-intensity exercise group

One of the investigators led the high-intensity exercise group. High-intensity interval training (HIIT) is a medium for physical activity that utilizes short bursts of intense exertion intertwined with brief rest breaks to achieve a moderate-vigorous workout quickly (Deka et al., 2022; Hwang et al., 2019). High-intensity interval training was implemented as an exercise intervention due to the high performance on the TUG and additional positive effects on physical and mental health in the older adult population (Hwang et al., 2019). Exercises provided to the participants included seated punches, standing marches, mini squats, arm circles, standing hamstring curls, and a modified jumping jack. The participants were shown each exercise, and then immediately practiced with the investigator, who provided visual and manual feedback as needed. Each exercise was performed for 45 s and had 15 s of rest. The entire exercise program was repeated three times.

Please see Appendix G for the high-intensity exercises provided to the participants.

### 2.8 Program design and low-intensity exercise group

Low-intensity exercises can be considered any activity level performed below a perceived exertion of <55% of a person’s heart rate reserve (Izquierdo et al., 2021). Incorporating movements such as low-intensity exercise is needed in person’s who are deconditioned which is a common occurrence due to the sedentary predisposition of this population. These types of exercises are multimodal allowing for improvements in strength and balance while also improving compliance to a structured exercise plan (Fyfe et al., 2022; Carraro et al., 2018; Flairty and Scheadler, 2020). The participants were shown each exercise, and then immediately practiced with the investigator, who provided visual and manual feedback as needed. The exercises included bed mobility, bridges, single leg raises, knee extension, scapular retraction with use of a resistance band, and mini squats with assistance. Each exercise was performed for 10–12 repetitions × 2–3 sets.

Please see Appendix F for the low-intensity exercises provided to the participants.

After both groups had completed their exercises, the participants were asked to complete the post-confidence survey. The post-confidence survey included the same questions as the pre-confidence survey and allowed the investigators to see if a change occurred after the course was provided.

Descriptive analysis was performed in Excel Microsoft Office software, version 16.87 (Redmond, Washington, United States).

## 3 Results: details to understanding program elements

### 3.1 Quantitative and qualitative data

Thirty-eight participants completed the course provided, and data were collected from 29 of them. The sample consisted of 25 females and four males, with an average age of 76.1 years, who reported exercising an average of 2.8 days per week. Seventy-four percent of this sample reported a fall in which they unintentionally came to rest on the ground or onto another lower level (MMWR, 2006). Of those who reported having a fall in the last year, 24% said that their fall (s) occurred outside. Eighty-three percent of the participants reported not using any assistive device for ambulation.

Please see Appendix H Tables A1–A7 for the Results of the Demographic Survey.

### 3.2 Pre-course confidence survey results and dropouts

Before the course began, 9% of the participants strongly agreed that they knew how to lower their risk for metabolic syndrome, 18% strongly agreed they knew how to manage their stress and anxiety, and 23% strongly agreed they knew how to move safely with exercise. Additionally, 73% strongly agreed that they understood the importance of exercise for their health and well-being. Seven participants dropped out due to prior time commitments conflicting with the class schedule, and their data was not included in the confidence survey calculations.

### 3.3 Post course confidence survey results

Questions from the confidence survey measured the participants’ confidence in their knowledge regarding metabolic syndrome, management of stress and anxiety, and the importance of physical activity.

Regarding question one, “I know how to lower my risk of metabolic syndrome,” before the course began, 73% of the participants strongly disagreed, disagreed, or were neutral regarding their confidence in lowering the risk of metabolic syndrome. After the course, 82% of the participants agreed or strongly agreed with knowing how to reduce the risk of metabolic syndrome.

Regarding question two, “I know how to manage my stress and anxiety,” before the course began, 41% of the participants strongly disagreed, disagreed, or were neutral about their confidence in managing stress and anxiety. After the course, 91% of the participants agreed or strongly agreed with knowing how to manage stress and anxiety.

Regarding question three, “I know how to move safely with exercise,” 37% of the participants strongly disagreed, disagreed, or were neutral about their confidence in moving safely with exercise. After the course, 96% of the participants agreed or strongly agreed with knowing how to move safely with exercise.

Regarding question four, “I know that exercise is needed for my health and well-being,” 9% of the participants strongly disagreed, disagreed, or were neutral about their confidence in knowing the importance of exercise. After the course, 95% of the participants agreed or strongly agreed with exercise being needed for health and well-being.

Please see Appendix I Tables A9, 10 for the Results of the Confidence Surveys.

## 4 Discussion

### 4.1 Practical implications

The purpose of this community case study was to measure the impact of education along with prescription of tailored exercise for management of metabolic syndrome in the older adult population. The investigators hypothesized that combining education, mental health practices, and personalized physical exercises would improve confidence levels in older adults.

Providing education, mental health practices, and high-intensity or low-intensity exercise interventions allowed an increased perceived confidence score in older adults. This was shown in questions regarding lowering the risk for metabolic syndrome and performing mental and physical exercises safely. Despite having a short timeframe to provide education and exercise, the survey results showed absolute changes in the “strongly agree” responses after the interventions were given. Specifically, confidence findings improved in knowing how to lower the risk of metabolic syndrome, which increased by 46%; how to manage stress and anxiety, which increased by 50%; and how to exercise safely, which increased by 41%. In summary, the confidence scores indicated that older adults knew the importance of physical activity for their physical and psychological health. However, after the course was provided, confidence was found in how to lower the risk for metabolic syndrome, manage stress and anxiety, and move safely with exercise. The data collected demonstrates that education combined with tailored exercise prescription impacts confidence scores in this population.

### 4.2 Limitations

There were limitations in this study. The sample size was small, and the recruited participants had a misconception of their expectations for the session initially which led to some frustration when explaining the research study. Additionally, the transition from the educational presentation to the intervention was time-consuming. The participants needed to relocate from the presentation room to the gym which allowed time to be lost and some participants to leave the study due to other time constraints. Another limitation was that this sample of participants might not represent a typical population treated by healthcare providers. The demographic results indicate that the participants, mainly community ambulators, were relatively active. In addition, the group was primarily Caucasian (26 out of 29 participants) and female (25 female, four male). The group also demonstrated a high level of independence in that they drove themselves to the facility and were fully independent in their performance of activities of daily living. Therefore, participants did not represent a diverse demographic group and only consisted of paying fitness center members who attended it regularly.

## 5 Conclusion

### 5.1 Lessons learned and future applications

Educating older adults on the importance of daily movement per their fitness level while recognizing the signs of metabolic syndrome can be essential for minimizing the effects of a sedentary lifestyle and promoting functional independence. Additionally, improved confidence levels like what was seen in this study can lead to self-empowerment and encourage the independent performance of daily activities. This study raised participants’ awareness of a potentially debilitating condition while also allowing an enhanced knowledge of risk-reduction methods. Implementing this course in a clinical setting would require minimal time while creating lasting changes for individuals. These impactful changes include reducing personal and healthcare system costs, as well as enhancing quality of life. Additional studies should further investigate the long-term effects of education combined with individualized exercises but in a larger and diverse population.

## Data Availability

The original contributions presented in the study are included in the article/Supplementary material, further inquiries can be directed to the corresponding author.
