# Supplementary material for: The importance of education combined with tailored exercise in the health and wellness of older adults: a community case study
Source: Front Psychol. 2024 Oct 10;15:1488903. doi: 10.3389/fpsyg.2024.1488903 (PMC11499106; doi:10.3389/fpsyg.2024.1488903)
Supplement: Supplementary file 1 [file Table_1.DOCX]

Appendix A

*Informed Consent Form*


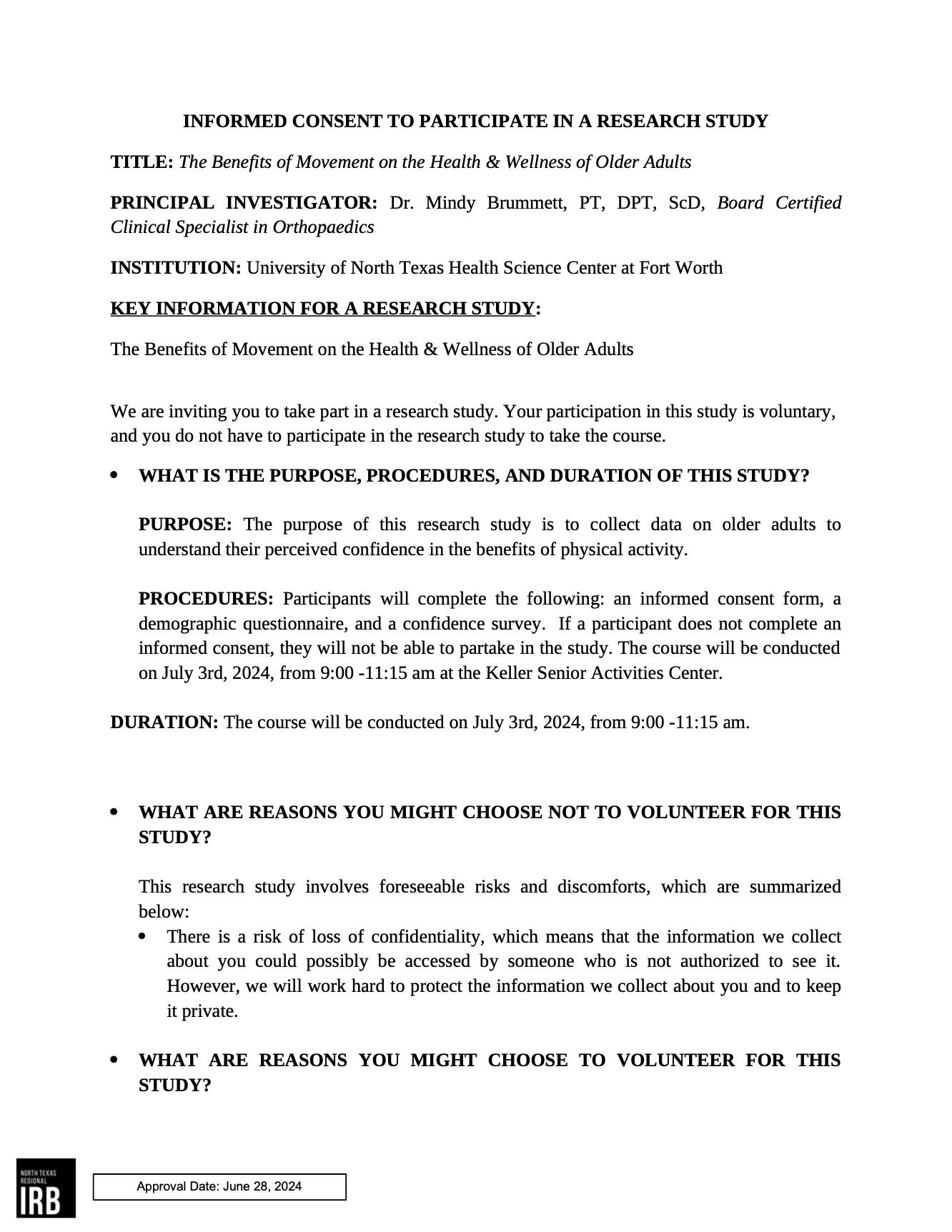


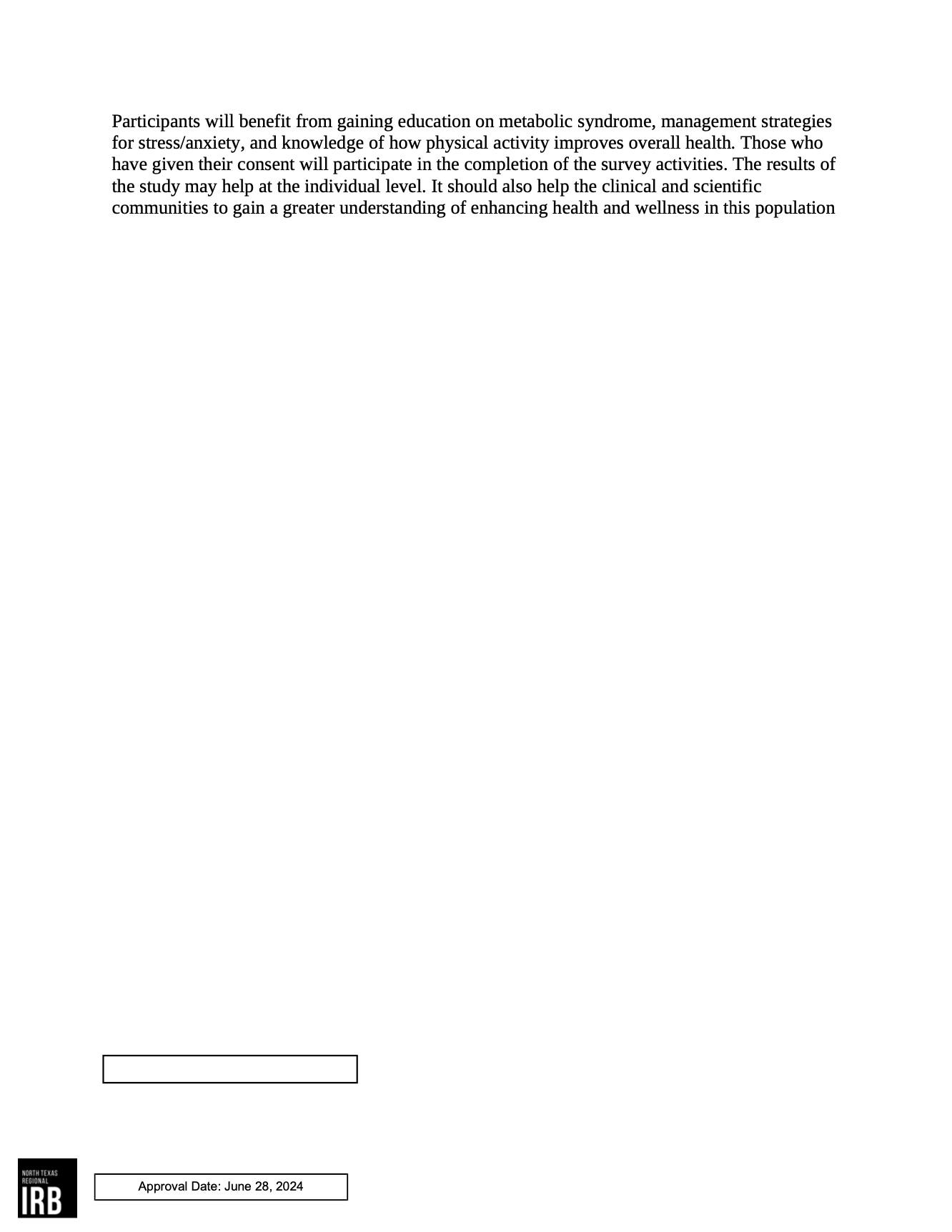

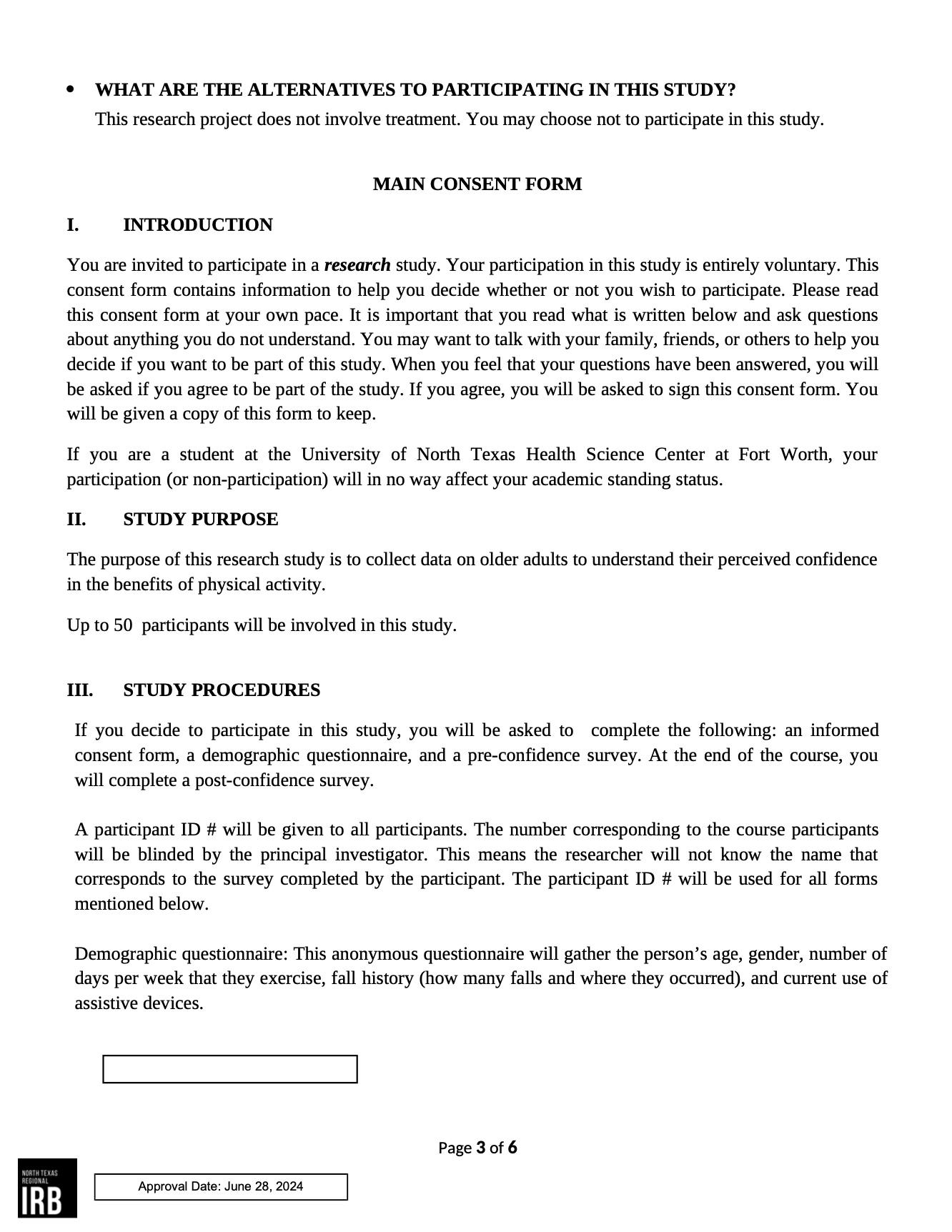

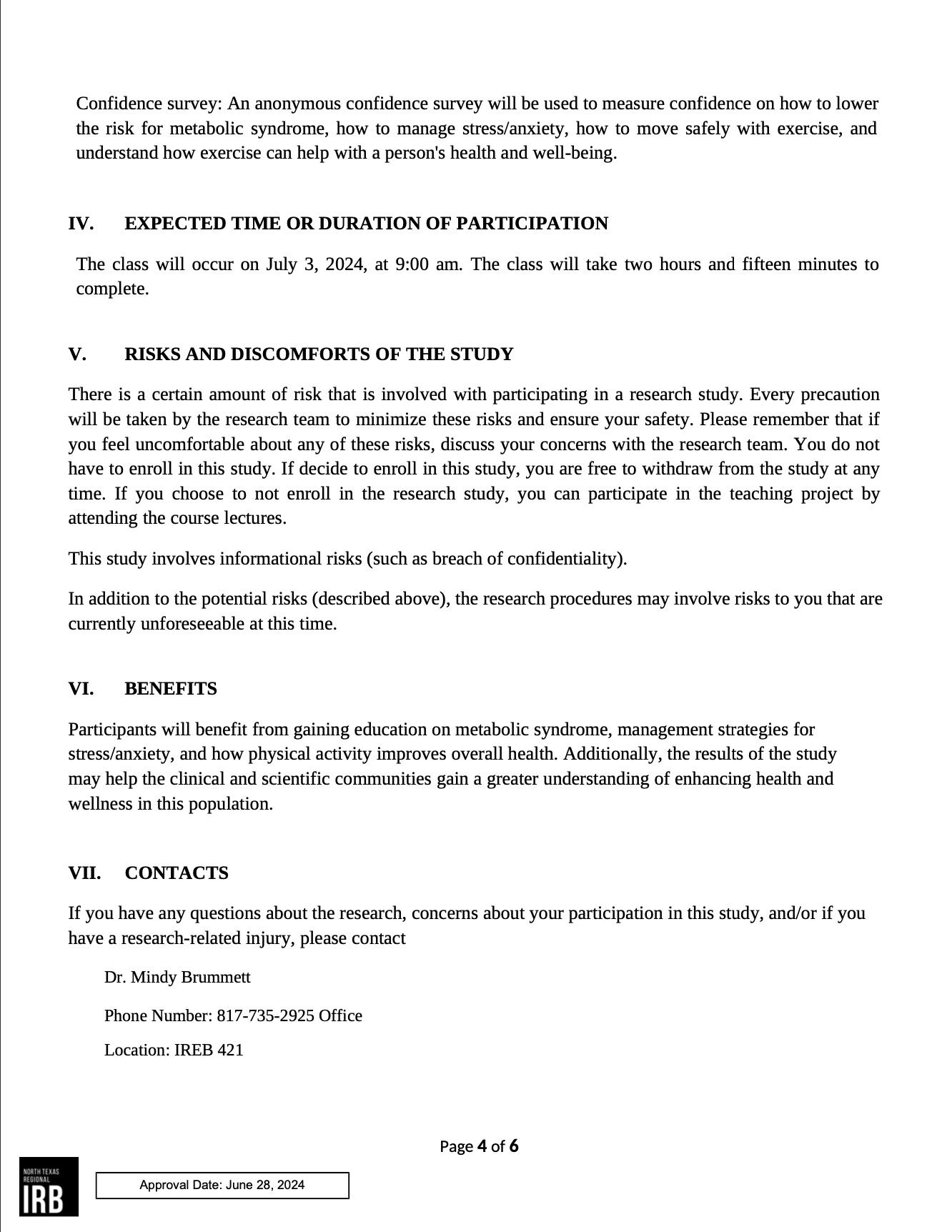

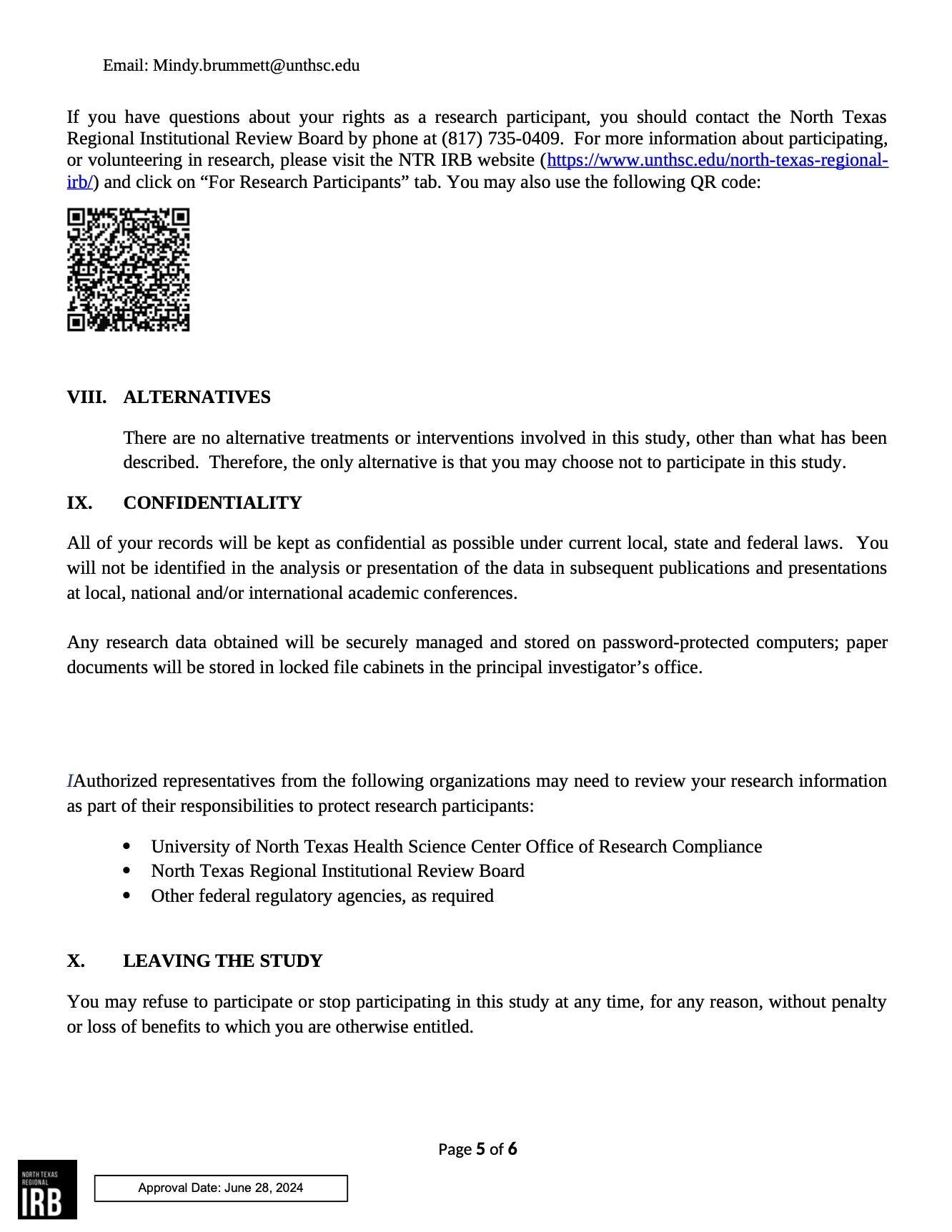

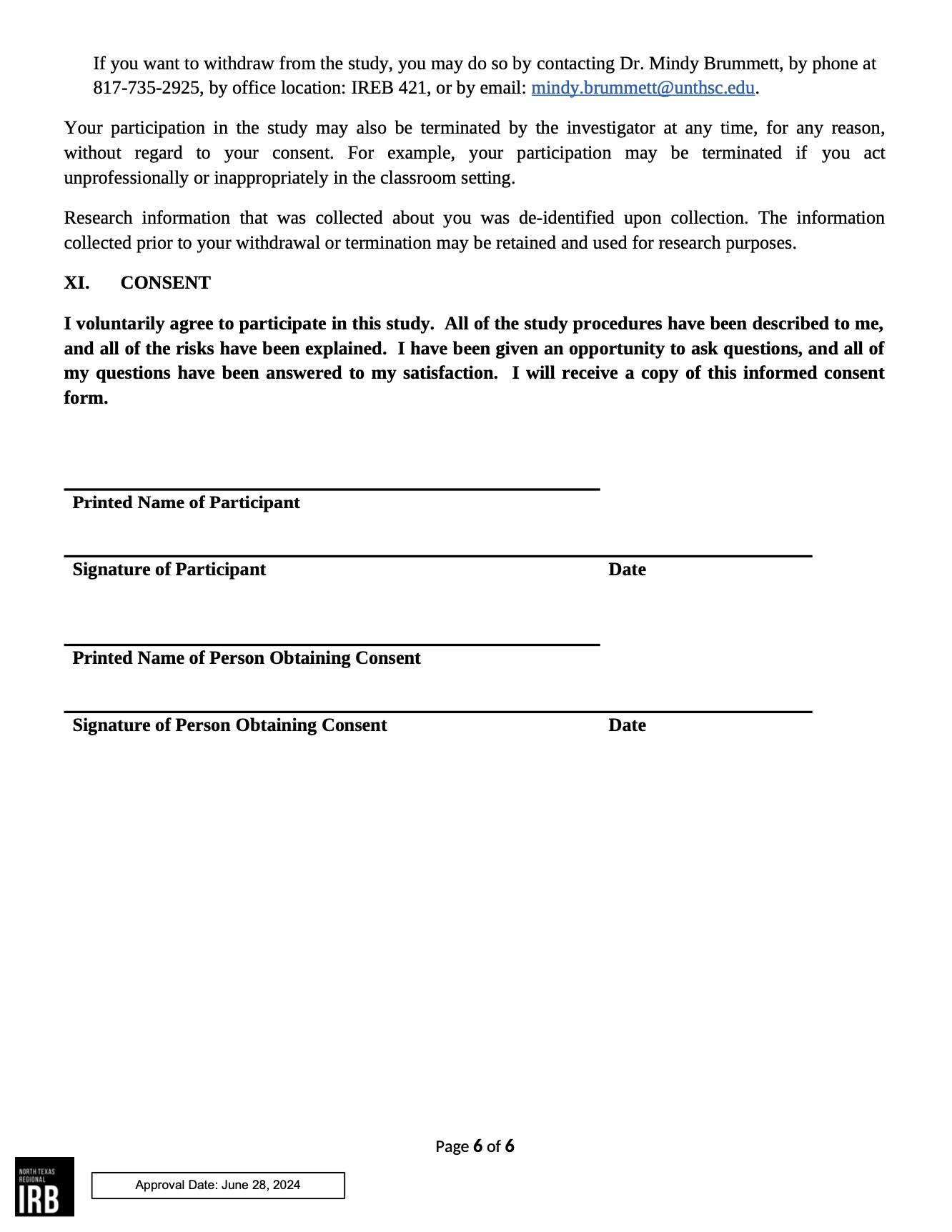


Appendix B

*Demographic Questionnaire*


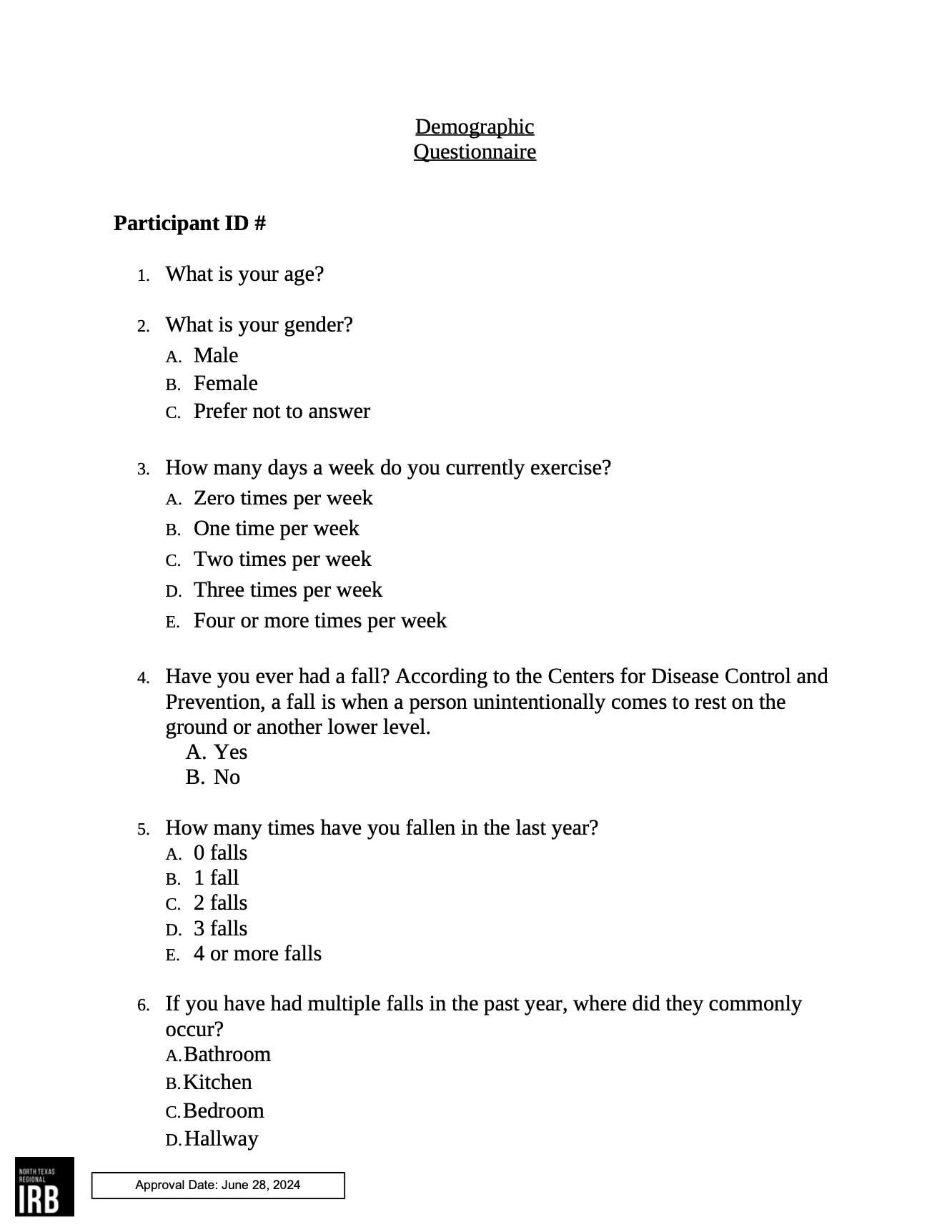


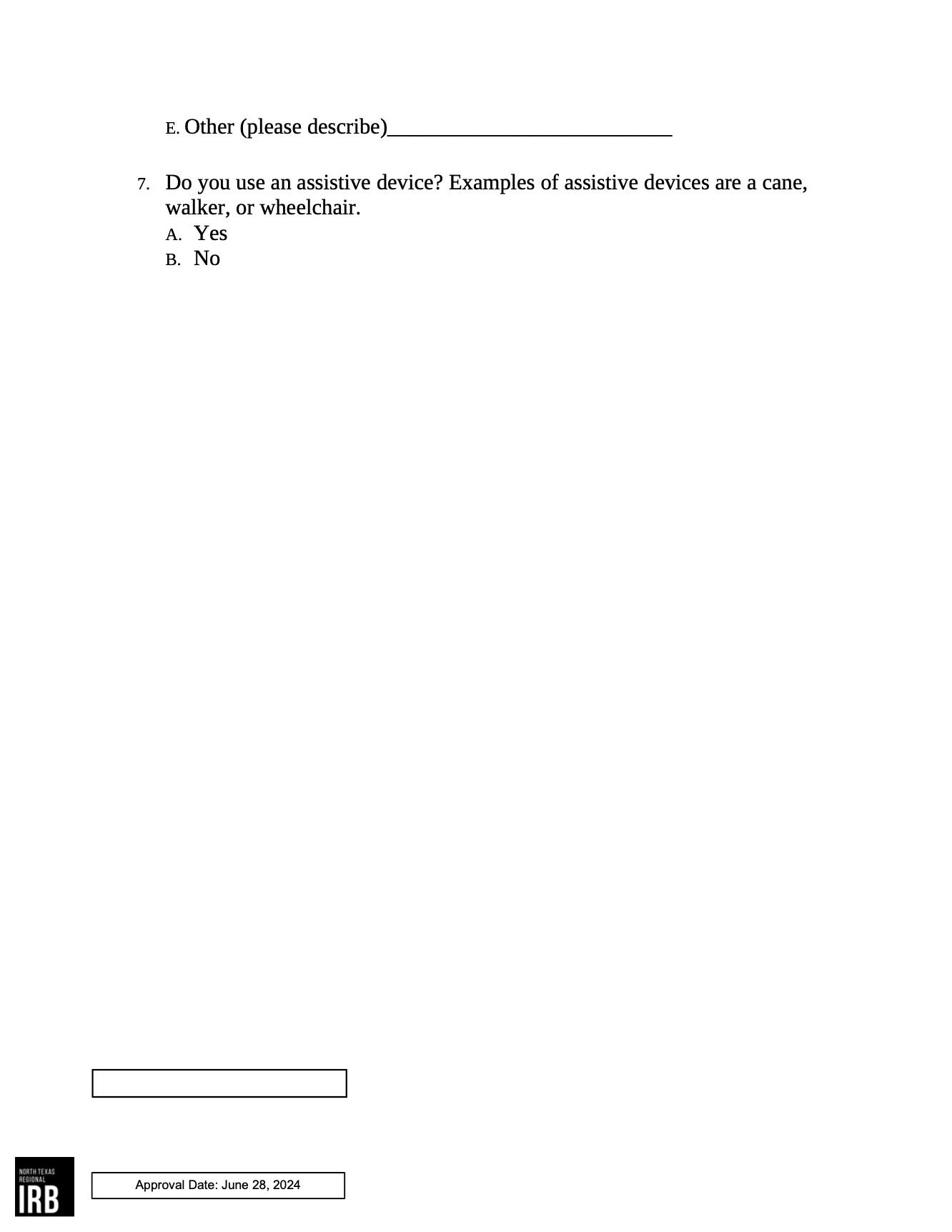


Appendix C

*Educational Presentation Slides*


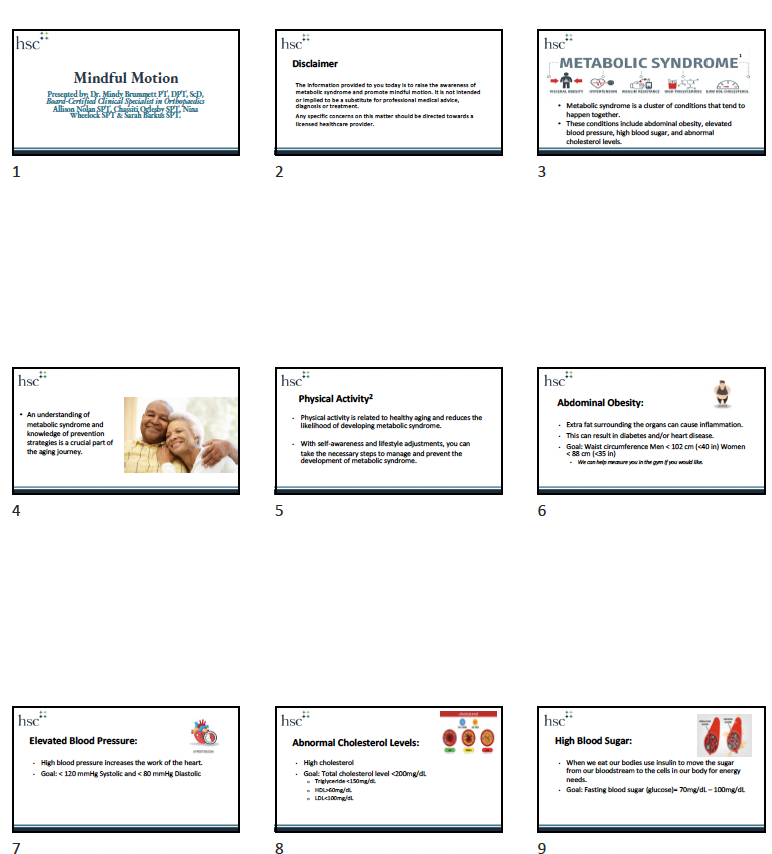


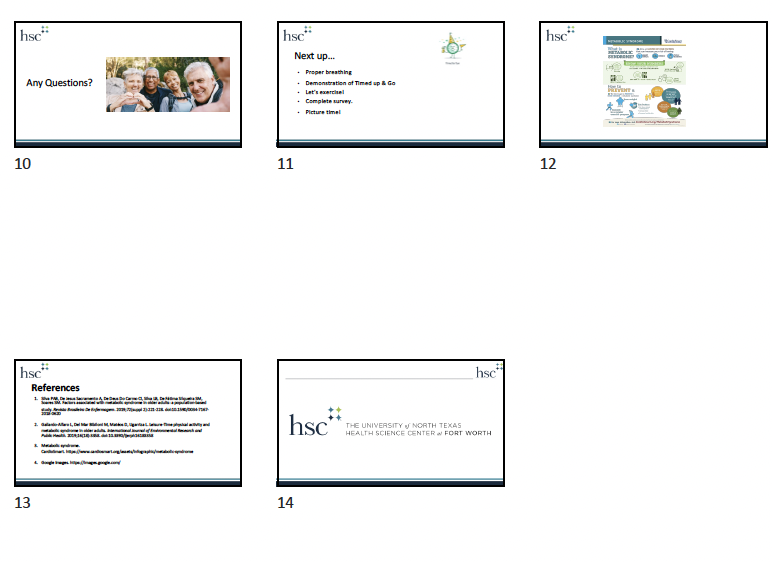


**Appendix D**

Confidence Survey


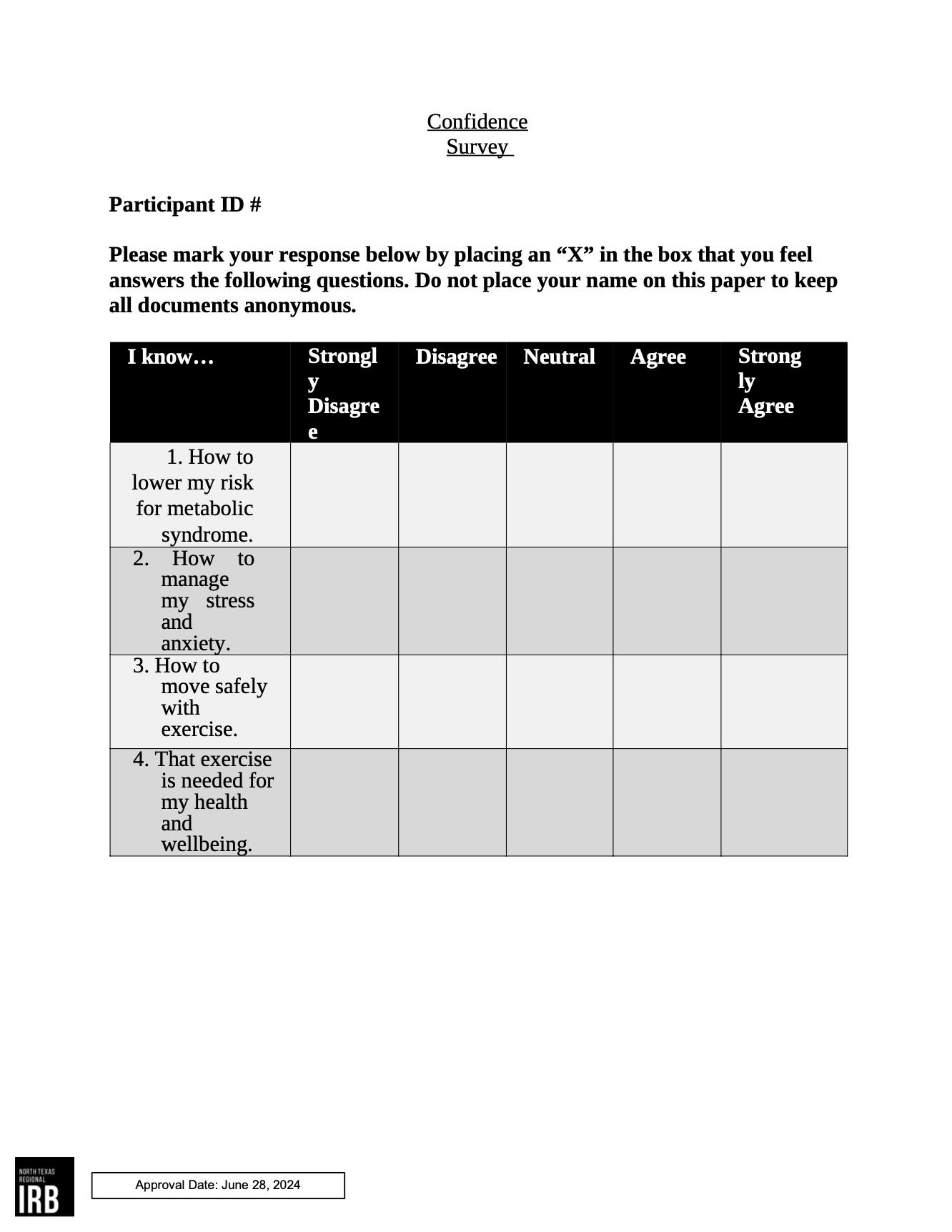


Appendix D

*Mental Health Exercise Handout*

*
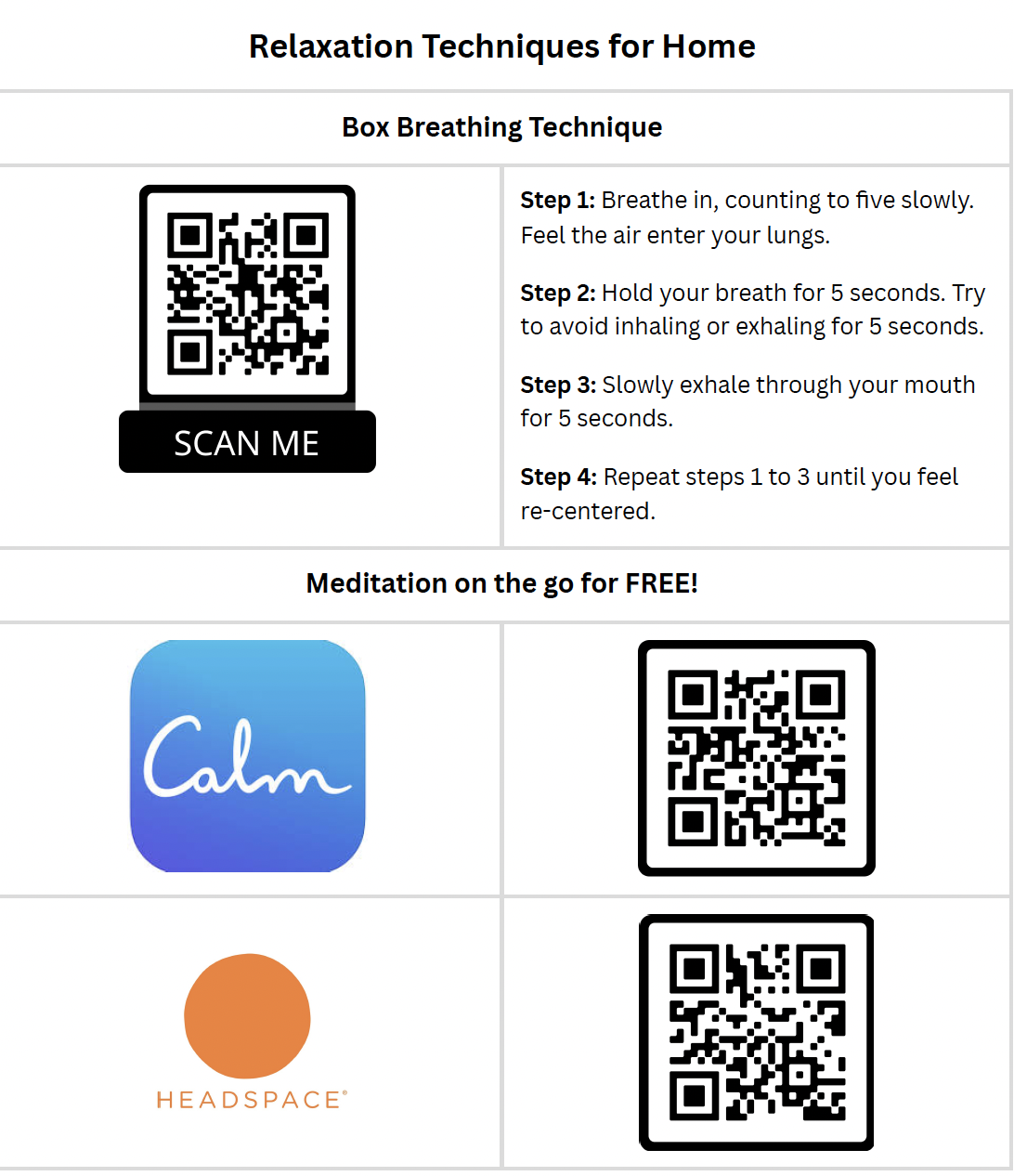
*

Appendix G

*High-Intensity Exercise Handout*

*
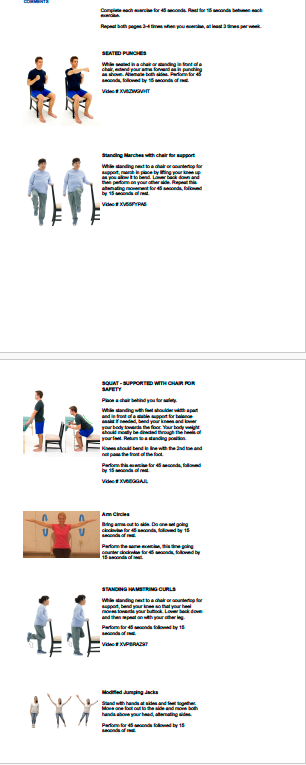
*

Appendix F

*Low-Intensity Exercise Handout*

*
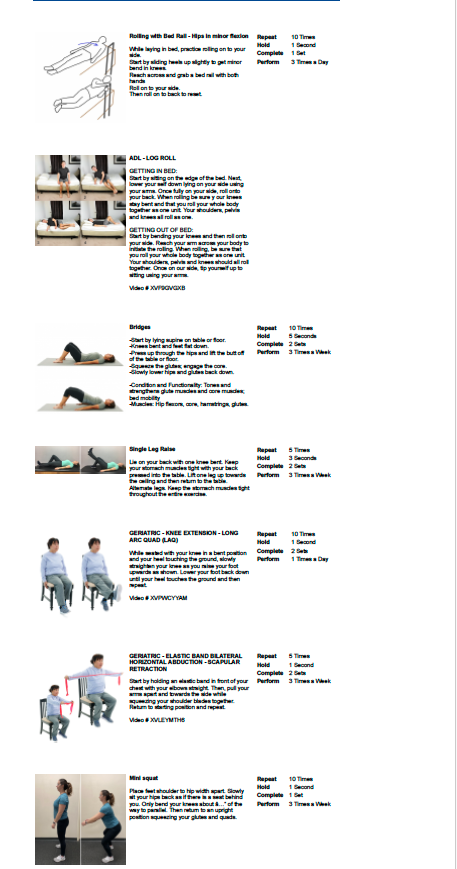
*

Appendix G

*Pictures From Senior Center*


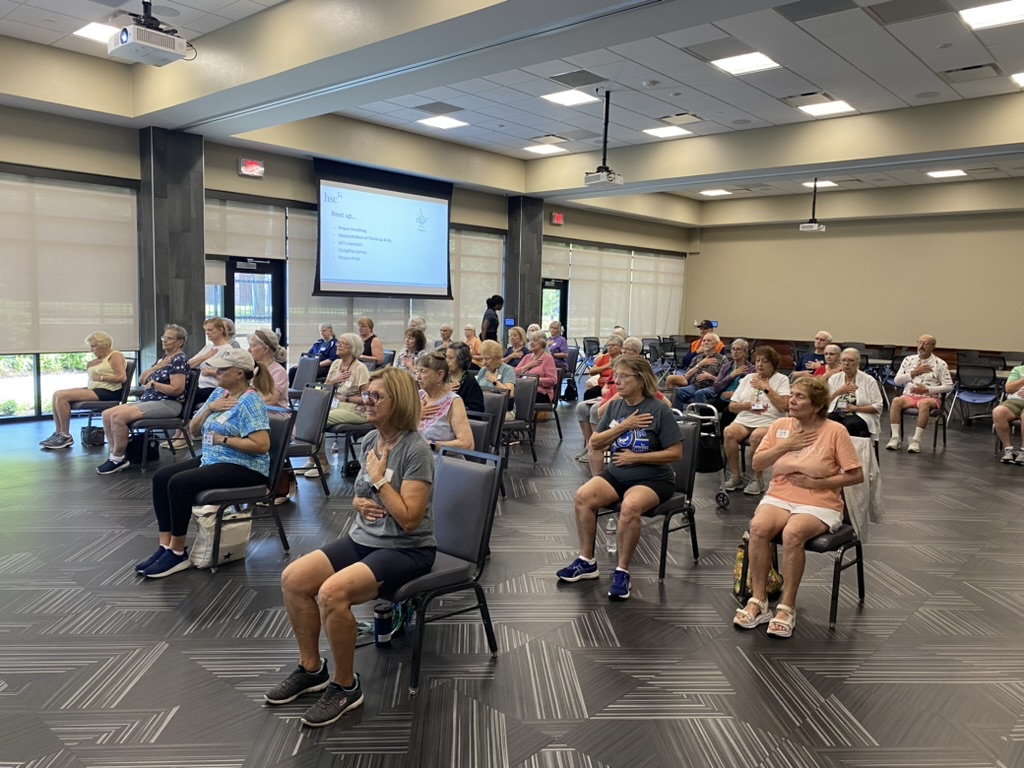


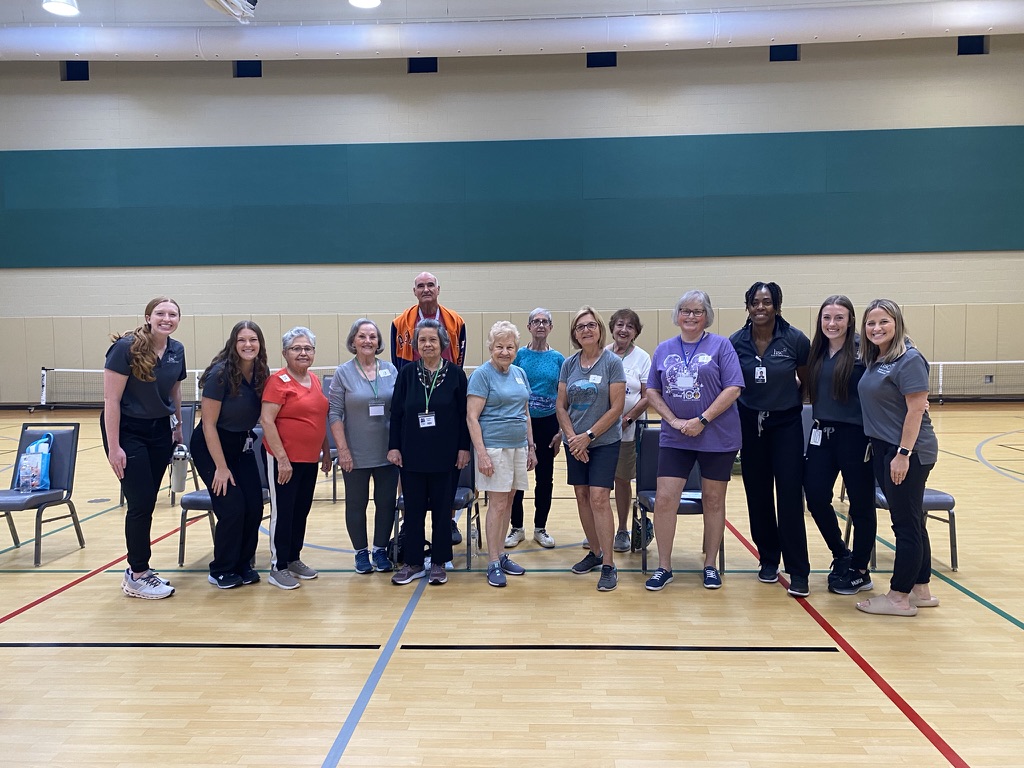


Appendix H

Demographic Questionnaire Results

**Table A1.** Age.

| Average age—76 years old |
| --- |
| SD ± 6.4 years |

**SD: standard deviation**

**Table A2.** Gender.

| **Gender** | **% of Sample** |
| --- | --- |
| Male | 13.8 |
| Female | 86.2 |

**Table A3.** Days per week of exercise.

| **How Many Days a Week Do You Exercise?** | **% of Sample** |
| --- | --- |
| 0 | 6.9 |
| 1 | 10.3 |
| 2 | 13.8 |
| 3 | 31.0 |
| 4+ | 37.9 |
| Average: 2.8 days | |

**Table A4.** Falls.

| **Have You Ever Fallen?** | **% of Sample** |
| --- | --- |
| Yes | 72.4 |
| No | 27.5 |

**Table A5.** Falls per year.

| **How Many Falls in One Year?** | **% of Sample** |
| --- | --- |
| 0 | 58.6 |
| 1 | 13.8 |
| 2 | 6.9 |
| 3 | 13.8 |
| 4+ | 6.9 |
| **Average: 0.97 falls per year**  **SD: 1.375** | |

**SD=Standard deviation**

**Table A6.** Location of the fall.

| **Where Did The Fall Occur?** | **% of Sample** |
| --- | --- |
| Outside | 24 |
| Kitchen | 6.9 |
| Other | 6.9 |
| Bathroom | 6.9 |
| Bedroom | 3.4 |
| Hallway | 3.4 |

**Table A7.** Assistive device use.

| **Do You Use an Assistive Device?** | **% of Sample** |
| --- | --- |
| Yes | 17.2 |
| No | 82.8 |

Appendix I

*Confidence Survey Results*

**Table A8.** Confidence survey results acknowledging confidence in lowering metabolic syndrome risks (number of participants and percent of sample).

| **Ranking Scale** | **Question 1 Pre-Course** | **Question 1 Post-Course** | **Absolute % Change** |
| --- | --- | --- | --- |
| Strongly Agree | 2 (9%) | 12 (55%) | 46% |
| Agree | 4 (18%) | 6 (27%) | 9% |
| Neutral | 7 (32%) | 3 (14%) | 18% |
| Disagree | 5 (23%) | 0 (0%) | 23% |
| Strongly Disagree | 4 (18%) | 1 (8%) | 10% |

Question 1: I know how to lower my risk for metabolic syndrome.

**Table A9.** Confidence survey results acknowledging confidence in managing stress and anxiety (number of participants and percent of sample).

| **Ranking Scale** | **Question 2 Pre-Course** | **Question 2 Post-Course** | **Absolute % Change** |
| --- | --- | --- | --- |
| Strongly Agree | 4 (18%) | 15 (68%) | 50% |
| Agree | 9 (41%) | 5 (23%) | 18% |
| Neutral | 6 (27%) | 1 (5%) | 22% |
| Disagree | 2 (9%) | 1 (5%) | 4% |
| Strongly Disagree | 1 (5%) | 0 (0%) | 5% |

Question 2: I know how to manage my stress and anxiety.

**Table A10.** Confidence survey results acknowledging confidence in safe physical activity (number of participants and percent of sample).

| **Ranking Scale** | **Question 3 Pre-Course** | **Question 3 Post-Course** | **Absolute % Change** |
| --- | --- | --- | --- |
| Strongly Agree | 5 (23%) | 14 (64%) | 41% |
| Agree | 9 (41% | 7 (32%) | 9% |
| Neutral | 5 (23%) | 1 (5%) | 18% |
| Disagree | 2 (9%) | 0 (0%) | 9% |
| Strongly Disagree | 1 (5%) | 0 (0%) | 5% |

Question 3: I know how to move safely with exercise.

**Table A11.** Confidence survey results acknowledging confidence in exercise helping health and well-being (number of participants and percent of sample).

| **Ranking Scale** | **Question 4 Pre-Course** | **Question 4 Post-Course** | **Absolute % Change** |
| --- | --- | --- | --- |
| Strongly Agree | 16 (73%) | 17 (77%) | 4% |
| Agree | 4 (18%) | 4 (18%) | 0% |
| Neutral | 0 (0%) | 1 (5%) | 5% |
| Disagree | 0 (0%) | 0 (0%) | 0% |
| Strongly Disagree | 2 (9%) | 0 (0%) | 9% |

Question 4: I know that exercise is needed for my health and well-being
